# The indispensable role of mammalian iron sulfur proteins in function and regulation of multiple diverse metabolic pathways

**DOI:** 10.1007/s10534-019-00191-7

**Published:** 2019-03-28

**Authors:** Tracey A. Rouault

**Affiliations:** National Institutes of Health, Bethesda, MD 20892 USA

**Keywords:** Iron sulfur proteins, IRP1 and IRP2, ABCB7 and Atm1, CIAO1, LYR motif, HSC20

## Abstract

In recent years, iron sulfur (Fe–S) proteins have been identified as key players in mammalian metabolism, ranging from long-known roles in the respiratory complexes and the citric acid cycle, to more recently recognized roles in RNA and DNA metabolism. Fe–S cofactors have often been missed because of their intrinsic lability and oxygen sensitivity. More Fe–S proteins have now been identified owing to detection of their direct interactions with components of the Fe–S biogenesis machinery, and through use of informatics to detect a motif that binds the co-chaperone responsible for transferring nascent Fe–S clusters to domains of recipient proteins. Dissection of the molecular steps involved in Fe–S transfer to Fe–S proteins has revealed that direct and shielded transfer occurs through highly conserved pathways that operate in parallel in the mitochondrial matrix and in the cytosolic/nuclear compartments of eukaryotic cells. Because Fe–S clusters have the unusual ability to accept or donate single electrons in chemical reactions, their presence renders complex chemical reactions possible. In addition, Fe–S clusters may function as sensors that interconnect activity of metabolic pathways with cellular redox status. Presence in pathways that control growth and division may enable cells to regulate their growth according to sufficiency of energy stores represented by redox capacity, and oxidation of such proteins could diminish anabolic activities to give cells an opportunity to restore energy supplies. This review will discuss mechanisms of Fe–S biogenesis and delivery, and methods that will likely reveal important roles of Fe–S proteins in proteins not yet recognized as Fe–S proteins.

## Mammalian Fe–S proteins were largely overlooked until recent years

**Fig. 1:**
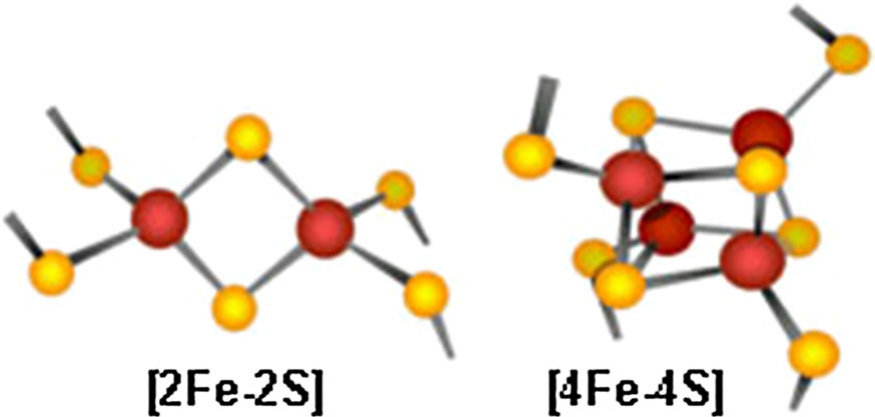
Two common types of Fe–S clusters, with sulfur represented by yellow spheres and iron represented by red spheres. Here, the cysteinyl sulfurs of proteins that ligate the clusters are shown on the outer margin of each cluster. (Color figure online)

Fe–S proteins were not discovered and characterized until the late 1950s and early 1960s when methods were developed that detected their intrinsic magnetic properties, including electron paramagnetic imaging and Mossbauer spectroscopy methods (reviewed in Beinert et al. 1997). Fe–S clusters are composed of inorganic iron and sulfide atoms that assemble in different stoichiometries, including those composed of two iron and two sulfur atoms, and the common cubane cluster composed of four iron and four sulfide atoms; Fe–S clusters generally ligate to proteins through iron–cysteinyl bonds. Much more complex clusters are required for the functions of nitrogenase, which is key to nitrogen fixation, and hydrogenase, which releases energy upon oxidation of hydrogen. The fact that the characteristic absorbance of Fe–S clusters occurs in a portion of the uv visible spectrum where many other materials absorb permitted them to remain relatively unnoticed long after other key cofactors such as heme were identified and studied (Beinert 2000). Other factors that contributed to their obscurity include that they are readily disassembled by exposure to oxygen and other oxidants, and they are often degraded by the time a protein of interest has been isolated for further characterization. (Fig. 1)

## Discovery of the Fe–S cluster of cytosolic aconitase and its role in regulation of cytosolic iron metabolism

Before 1990, very few Fe–S proteins had been identified and studied in mammalian cells, though many more had been discovered in bacteria (Johnson et al. 2005). They included two enzymes of the citric acid cycle, succinate dehydrogenase, and mitochondrial aconitase, which was extensively characterized by Beinert and colleagues in the 1980s (Kennedy et al. 1983; Beinert and Kennedy 1993). Complex I was also known to contain multiple deeply buried Fe–S clusters, but it was otherwise widely assumed that Fe–S proteins had gradually been phased out of metabolism during evolution of higher life forms because of their intrinsic lability.

**Fig. 2:**
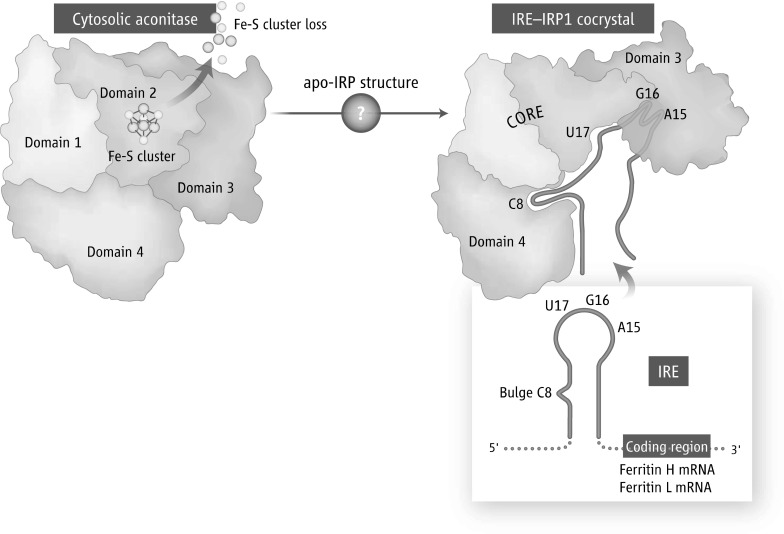
IRP1 alternates between function as a cytosolic aconitase, which contains a [Fe4–S4] cluster in the active-site cleft, to an apoprotein form that lacks the cluster and binds to IRE stem-loop structures present in several iron transcripts. Upon binding, IRP1 represses translation of multiple transcripts that contain IREs near the 5′-end of the transcript, including Ferritin H and L transcripts, HIF2 α, erythrocytic ALAS, and the iron export protein, ferroportin. Conversely, IRP binding to mRNAs that contain IREs at the 3′-UTR protects transcripts from endonucleolytic degradation: the most well-known such transcript is transferrin receptor 1. Apo-IRP1 undergoes a large conformational change that creates a complex IRE-binding pocket, in which the bulge C of the IRE binds to a pocket in domain 4, and three residues of the loop make finger-like binding projections into regions of domain 3 that become accessible after conformational change. The length of the upper base-paired stem of the IRE derived from NMR structural solution (Addess et al. 1997) optimizes the distance between the main IRP contact points, the unpaired C of the stem, and residues A15, G16 and U17 of the loop, resulting in high affinity binding Figure from Rouault (2006)

During studies of mammalian regulation of iron metabolism genes, my colleagues and I discovered that multiple transcripts of iron proteins were regulated by a system known as the IRE-IRP system, in which transcripts that contain an RNA stem-loop known as an iron-responsive element (IRE) bind cytosolic iron-sensing proteins now known as iron regulatory proteins 1 and 2 (IRP1 and IRP2) in iron-depleted cells (reviewed in Klausner et al. 1993). Upon affinity purification of IRP1 using a column to which IREs were bound, the protein now known as IRP1 was purified and cloned (Rouault et al. 1990) and sequence resemblance to mitochondrial aconitase, particularly in the active site residues, suggested that IRP1 represented a functional cytosolic aconitase that interconverted citrate and isocitrate (Kaptain et al. 1991), which was confirmed by direct purification of beef liver cytosolic aconitase (Kennedy et al. 1992). Multiple studies demonstrated that alternation of IRP1 between functioning as an RNA binding protein or as a cytosolic aconitase depended on whether an intact [4Fe–4S] cluster was ligated in the active site cleft where a single cluster iron ligated the enzymatic substrate for aconitase activity, or IRP1 was present as an apoprotein that was devoid of an Fe–S cluster. The Fe–S cluster was present in a solvent-exposed cleft of the enzyme and was experimentally shown to be highly vulnerable to degradation (Haile et al. 1992). Structures of the cytosolic aconitase (Dupuy et al. 2006) and apo-IRP1 bound to the IRE (Walden et al. 2006) revealed how the IRE achieves high affinity binding to apo-IRP1 by binding to sites rendered accessible by conformational changes that occur upon loss of binding of the Fe–S cluster to the active site cleft (Fig. 2). These studies then led to intense focus on identifying the mechanisms by which Fe–S clusters are assembled and delivered to recipient proteins in mammalian cells.

## Overlapping and distinct roles of Irp1 and Irp2 were revealed by genetic studies

To dissect the physiological roles of the two IRPs, which represented a duplicated gene pair in which both are apparently expressed in differing ratios in all cell types, genetic knockouts of each IRP were generated in mice and analyzed. Deletion of both IRPs was embryonic lethal at the blastocyst stage of development (Smith et al. 2006), emphasizing the indispensable physiological need for Irp function at very early stages of development. *Irp2*−*/*− mice displayed neurodegeneration which most adversely affected motor neurons (Jeong et al. 2011) and caused axonopathy and degeneration in multiple other brain regions (LaVaute et al. 2001). In addition, animals developed a mild anemia and erythropoietic protoporphyria (Cooperman et al. 2005; Galy et al. 2005) attributable to functional iron deficiency from loss of TfR1 mediated iron uptake and overexpression of the multimeric cytosolic iron sequestration protein, ferritin. The clinical presentation of neurodegenerative disease was subtle, and mice often lived for 12 months or more. A dispute about the phenotype (Galy et al. 2006) was resolved by generation of a third knockout mouse (Zumbrennen-Bullough et al. 2014) which also manifested iron misregulation and impaired motor functions (summarized in Ghosh et al. 2015).

The clinical presentation of the *Irp1*−*/*− mice differed, perhaps because Irp1 was the predominant Irp in many non-CNS tissues including fat and kidneys (Meyron-Holtz et al. 2004a). *Irp1*−*/*− mice developed polycythemia (Ghosh et al. 2013; Anderson et al. 2013) and died suddenly on low iron diets, likely from hemorrhages related to occlusive clot formation caused by high hematocrits, which were even higher on the low iron diet (Ghosh et al. 2013). The polycythemia was driven by de-repression of HIF2 α, which is encoded by an IRE-containing transcript (Sanchez et al. 2007) that is primarily regulated by Irp1, likely because high amounts of Irp1 are expressed in erythropoietin-producing cells (Ghosh et al. 2013). HIF2 α drives expression of erythropoietin, which leads to excess red cell production, and also to pulmonary hypertension (Ghosh et al. 2013). Recent studies have shown that a stable nitroxide fed to animals, TEMPOL, can suppress the polycythemia of Irp1−/− mice, and also has a salutary effect on mice that have a mutation that interferes with function of the VHL degradation complex responsible for HIF degradation (Ghosh et al. 2018).

Experimental studies showed that IRPs faithfully reproduce their physiological activities when cells are cultured at low oxygen concentrations comparable to those that prevail in animal tissues (3–10%), rather than at room air concentrations of 21%, which constitutes hyperoxia and may adversely affect generation of meaningful experimental results in many settings (Meyron-Holtz et al. 2004b).

## How are Fe–S clusters synthesized and ligated to recipient proteins in eukaryotic cells?

The discovery of bacterial operons that contained systems involved in synthesis of Fe–S clusters discovered in *Azotobacter vinelandii* and *E. coli* revolutionized studies of Fe–S biogenesis in cells. Bacterial operons generally contained the basic machinery for Fe–S cluster formation, consisting of a cysteine desulfurase that liberates sulfur for incorporation into nascent clusters (NifS or IscS), a scaffold protein upon which nascent clusters assemble (NifU or IscU), a ferredoxin which likely provides electrons for Fe–S cluster formation, and a chaperone-co-chaperone pair (HscA and B) that likely aid folding or cluster transfer (reviewed in Johnson et al. 2005). These genes are so highly conserved throughout evolution that counterparts were readily identified through homology searches in plants, fungi, and mammals (reviewed in Lill 2009; Rouault 2015a; Uzarska et al. 2018).

Based on studies performed in *S. cerevisiae*, it was proposed that all nascent Fe–S clusters were synthesized in the mitochondrial matrix, and a sulfur component was exported to cytosol by the ABC transporter, Atm1 (homologous to ABCB7 in mammals), whereupon specialized proteins in the cytosol, named the cytosolic iron sulfur assembly machinery or CIA machinery completed synthesis and delivery of Fe–S cofactors to numerous cytosolic and nuclear proteins (Lill et al. 2012), which include DNA metabolism proteins (Gari et al. 2012; Stehling et al. 2012), proteins involved in RNA metabolism (Barthelme et al. 2007), proteins involved in cytokinesis (Ben-Shimon et al. 2018) and in multiple other basic cellular processes.

The key role of Atm1 (ABCB7 in mammals) was postulated because it was alleged to be crucial for Fe–S acquisition of enzymatic function in a critical Fe–S enzyme of the cytosolic leucine biosynthetic pathway of yeast (Kispal et al. 1999), though this conclusion appears to be incorrect because a defect in the experimental design led to increased transcription of the entire leucine biosynthetic operon in cells that contained functional Atm1, because selective inactivation of Atm1 was achieved by using a plasmid that re-introduced functional Leu2 into a Leu2-null strain (reviewed in Rouault 2015a), and the comparison of leu 1 enzymatic activity that was performed on cells that contained inactivated Atm1 had markedly lower transcript levels of leu1, which would be expected to markedly decrease leu 1 protein levels and enzymatic activity in the cell line in which Atm1 function was knocked out using a construct that restored leu2 gene expression to the leu2 deficient strain. This flawed experimental design would make it unlikely that than the previously reported comparison of leu 1 enzymatic activity (Kispal et al. 1999) was made between cells with equal leu1 proteins levels (see supplementary info in Hausmann et al. (2008) for transcriptional data), and therefore does not support that Atm1 is needed for leu1 to acquire a cytosolic Fe–S cluster. Other supporting evidence that initial Fe–S formation occurred solely in mitochondria was that mitochondrial Fe–S proteins were described as fully functional in the Atm1 knockout strains, whereas cytosolic Fe–S proteins were alleged to not function if a critical Fe–S biogenesis component was not delivered by Atm1 (Kispal et al. 1999). However, mitochondrial Fe–S proteins were later judged to be more lacking in normal Fe–S cofactors than cytosolic proteins in the same yeast strains when rigorous biophysical methodologies were used for analysis (Miao et al. 2009), contradicting the argument that Fe–S assembly in the mitochondrial matrix was normal. Further experimentation in mammalian cells supports that mitochondrial Fe–S assembly is significantly negatively impacted by loss of the mammalian Atm1 counterpart, ABCB7 (Kim et al. 2018), whereas cytosolic Fe–S proteins are minimally affected.

## Dissecting the mechanism of Fe–S delivery to recipient proteins

An important breakthrough in general understanding occurred when the Fe–S protein, succinate dehydrogenase subunit B (SDHB) was discovered to bind directly to the cochaperone, HSC20, the mammalian orthologue of bacterial HscB in the Fe–S synthesis machinery. The yeast homologue of HSC20, Jac1, was already known to bind the yeast orthologues of the scaffold protein IscU, known as Isu1, and the orthologue of the mammalian chaperone HSPA9 (HscA in bacteria), Ssq1, from yeast studies (Andrew et al. 2006; Kampinga and Craig 2010). Dissection of the binding site of HSC20 to mammalian SDHB revealed that HSC20 contained a pocket-like domain that bound iterations of the motif, LYR, which was found at two different positions in the open reading frame of SDHB (Maio et al. 2014), in the SDHB assembly factor protein SDHAF1(Maio et al. 2016), in LYRM7, a protein involved in Fe–S acquisition of the single Fe–S cluster of respiratory complex III, and in numerous proteins of complex I. These discoveries led to the conclusion that a single adaptable cochaperone-scaffold complex delivers nascent iron-sulfur clusters to mammalian respiratory chain complexes I–III (Maio et al. 2017). The basic features of the LYR motif include an aliphatic residue, followed by a large aromatic (tyrosine or phenylalanine), followed by a positively charged arginine or lysine, which can be modeled to fit into a pocket of the solved structure of HSC20 (Maio and Rouault 2016; Bitto et al. 2008). Thus, the basic apparatus of the Fe–S biogenesis machinery has been defined for mitochondrial Fe–S biogenesis, though the roles of other involved proteins such as glutaredoxin 5 (GLRX5) (Ye et al. 2010), ISCA, a putative scaffold protein (Py et al. 2018) and multiple other proteins such as NFU (Tong et al. 2003) have not been specifically assigned, and may be involved as secondary scaffold carriers that deliver clusters to a subset of Fe–S proteins.

## Parallel systems for Fe–S synthesis in mitochondrial matrix and cytosolic/nuclear compartments of mammalian cells

In mammalian cells, a full complement of Fe–S biogenesis proteins is synthesized and targeted to the mitochondrial matrix, but almost all of these proteins have also been observed in the cytosolic/nuclear compartments of cells. Though they are often transcribed from the same gene, cytosolic forms are generated by alternative splicing (Tong and Rouault 2006), a weak mitochondrial targeting signal (Maio et al. 2014), alternative utilization of initiator AUGs that retained or skipped mitochondrial targeting information (Land and Rouault 1998) and possibly other mechanisms. These proteins have not been found in the cytosol of the model system *S. cerevisiae*, although the human cysteine desulfurase is clearly present and functional (Marelja et al. 2008, 2013). Unlike in bacterial systems, the cysteine desulfurase must be bound to another protein, ISD11 (also known as LYRM4) for function (Adam et al. 2006; Wiedemann et al. 2006; Shi et al. 2009).

## Complexes involved in synthesis of Fe–S clusters have been defined by native gels and mass spectrometry in both mammalian mitochondria and in the cytosolic/nuclear compartment

To better understand the process of Fe–S assembly, native gels and mass spectrometry have been helpful in defining stages in the assembly process. The validity of the approach was established by discovering that SDHB acquired its Fe–S clusters in stages, beginning with binding to HSC20 and ISCU, and progressing to ejection of the HSC20 transfer complex, transfer of the cluster to SDHB, binding of holo-SDHB to SDHA in the multiprotein SDHB complex, and finally to the membrane bound subunits, SDHC and SDHD (Maio et al. 2014). Unlike proposals for plants and yeast, the HSC20 transfer complex was shown to be required for Fe–S acquisition in complex I, which contains eight Fe–S clusters, and also in complex III, which contains a single [2Fe–2S] cluster (Maio et al. 2017). The universality of function suggested that each recipient protein possessed the requisite information that would allow the transfer apparatus to bind and transfer its cargo directly to the recipient proteins; these findings supported that the general underlying principles of Fe–S acquisition by recipient protein were shared. In some proteins such as lipoic acid synthase, which requires two Fe–S clusters, it is not yet clear exactly how other potential scaffolds participate in cluster transfer. They may mediate transfer as secondary scaffolds that contain targeting information for a specific subset and type of recipient proteins, such as those that contain Fe–S proteins that are consumed during incorporation of sulfur into octanoic acid to generate lipoic acid (6,8-dithiooctanoic acid), which functions in the swinging arm of multi- subunit dehydrogenases such as α-keto acid dehydrogenase, pyruvate dehydrogenase, branched chain ketoacid dehydrogenase and the glycine cleavage system (McCarthy and Booker 2017) (reviewed in Solmonson and DeBerardinis 2018) for which the exact molecular donor of the cluster is under study (McCarthy et al. 2018). It is likely that multiple secondary scaffolds may acquire their Fe–S cofactors from the canonical Fe–S biogenesis machinery, and may then distribute their bound Fe–S to specific subsets of proteins based on specific protein recognition sites in recipient proteins that promote complex formation and enshrouded transfer of Fe–S clusters from the secondary scaffold. An important role of NFU1 in lipoyl synthase cluster delivery was revealed in studies of human patients with Multiple Mitochondrial Dysfunctions Syndrome 1 (Navarro-Sastre et al. 2011; Cameron et al. 2011). The Cameron et al. study also revealed a critical role for BOLA3, a mitochondrial protein for which the role of Fe–S assembly has not yet been clarified.

Native gels and mass spectrometry also greatly enhanced studies of Fe–S biogenesis in the cytosol, where individual assembly steps have been defined (Kim et al. 2018). Differences between Fe–S biogenesis in the mitochondrial matrix include that the biogenesis machinery of the cytosol associates with Glutaredoxin 3 and BolA2, and instead of associating with a ferredoxin, the nascent complex may obtain necessary electrons through the thioredoxin system (thioredoxin reductase was identified in the complex), and/or from CBR1, an NADPH-Dependent Carbonyl Reductase that can bind glutathione (Malátková et al. 2010).

**Fig. 3:**
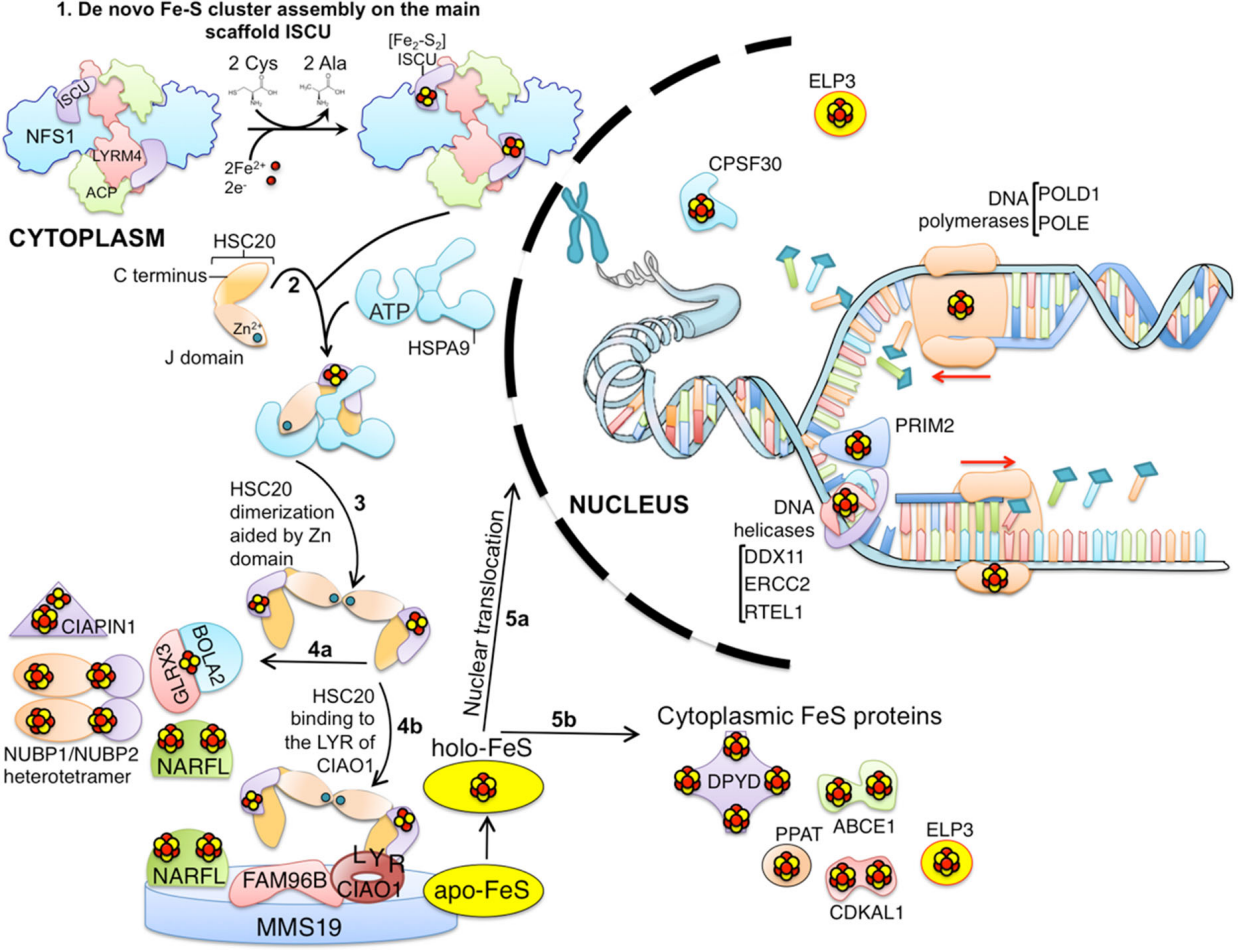
A summary of pathways of cytosolic Fe–S biogenesis. The nascent cluster assembles on the ISCU scaffold using cytosolic isoforms of biogenesis proteins, and interaction with the chaperone/co-chaperone apparatus permits these clusters to transfer directly to NARFL and the NUBP1/NUBP2 complex. A subset of Fe–S proteins involved in DNA metabolism and ribosome biogenesis, including DNA repair, RNA metabolism, and numerous other cytosolic proteins obtain their Fe–S cluster cofactors after interacting with the CIA complex, depicted as composed of CIAO1, MMS19, and FAM96B. Figure modified from Kim et al. (2018)

Studies showed that a branch point was reached in Fe–S cytosolic delivery to Fe–S proteins. In some examples, such as NUBP1, no other steps were required to facilitate transfer from the nascent apparatus, composed of the cysteine desulfurase, primary scaffold, and a chaperone co-chaperone pair. Interestingly, frataxin, which plays a critical function in the mitochondrial Fe–S biogenesis complex (Rouault 2015a), was clearly identified in the cytosol through mass spectrometry, though endogenous frataxin had not previously been found in mammalian cytosol. Cytosolic HSC20 was shown to be required for acquisition of radiolabeled iron in the DNA metabolism protein, POLD1, and for activation of Dihydropyrimidine Dehydrogenase in thymine metabolism (Kim et al. 2018) in experiments in which either HSC20 or ISCU were rendered dysfunctional. In some experiments, HSC20 expression was knocked out, and in others, the cytosolic isoform of the main scaffold protein ISCU was mutagenized and overexpressed to generate a dominant negative protein that was unable to release its nascent radiolabeled Fe–S cluster to cytosolic targets. Notably, these findings are inconsistent with the notion that these cytosolic Fe–S proteins derive their Fe–S co-factors solely from a precursor in the mitochondrial matrix (Lill 2009), since an exclusively cytosolic form of IscU was used in these experiments. In some other cytosolic and nuclear Fe–S proteins, the nascent complex associated with the large protein, CIAO1 (Srinivasan et al. 2007), by binding through HSC20 to an LYR sequence in CIAO1 (Kim et al. 2018). Since HSC20 dimerizes, it could readily bind to CIAO1 through one monomer, and bind to other binding partners through the LYR of the second half of the dimer in a cytosolic Fe–S transfer complex composed of MMS19 and FAM96B (Stehling et al. 2012; Gari et al. 2012). The CIA complex also contains other sites that may bind recipient proteins (Odermatt and Gari 2017) and it is particularly important for Fe–S acquisition by DNA metabolism proteins. Its role in Fe–S acquisition of other cytosolic proteins is a subject of ongoing work. However, recent work strongly indicates the CIA pathway previously outlined (Lill 2009) that originates solely in mitochondria is not the sole source of Fe–S to cytosolic recipients (Fig. 3).

## Implications for discovery of new Fe–S proteins

Discovery of the important role for LYR motifs opens the possibility that informatics can be used to identify prospective Fe–S proteins, and feasible follow-up steps including analysis by ICP-MS followed by scale up for analysis by definitive techniques that require large amounts of protein such as EPR and Mossbauer could revolutionize Fe–S protein discovery and lead to revision of many metabolic pathways (Rouault 2015a, b). The LYR motif has also been implicated as important in binding the acyl carrier protein (ACP) (Van Vranken et al. 2018) which may aid in coordinating respiration with fatty acid synthesis, though mutagenesis studies have not been performed to test the specific role of the LYR motifs in the larger binding site of ACP. The fact that numerous mutations of the LYR motif have been shown in experimental systems to prevent binding of the chaperone HSC20 (Maio et al. 2014, 2017; Maio and Rouault 2016) indicates that LYR motifs are involved in binding HSC20, but they may also bind ACP. These events need not be mutually exclusive, as the role of HSC20 binding to LYR could be crucial to Fe–S delivery, and after the Fe–S containing protein separates from the nascent Fe–S delivery machinery, it could bind a new binding partner such as ACP that possibly contributes to regulation of respiration. ACP was found in newer crystal structures of the nascent Fe–S complex (Cory et al. 2017; Boniecki et al. 2017), and its cytosolic ACP counterpart, fatty acid synthase (FASN), was identified by mass spectrometry in a cytosolic complex purified using antibodies to NFS1 in cytosolic fractions FASN (Kim et al. 2018).

## Role of mutations of Fe–S biogenesis machinery in multiple rare diseases

In a fundamental process such as Fe–S biogenesis, it is not surprising that mutations to the proteins involved in the process cause human diseases, particularly in cases when loss of function is not complete. The most well-known of these diseases is Friedreich’s ataxia, which causes ataxia and cerebellar dysfunction due to a loss of expression of frataxin, which is caused by by expansion of a trinucleotide repeat in the first intron that represses expression by an unknown mechanism. These diseases may offer insights into the mechanisms that account for tissue-specific pathology in disease. The subject has been extensively reviewed (Rouault and Tong 2008; Rouault 2012; Stehling et al. 2014), and many insights and potential therapeutic strategies may arise from better understanding the pathogenesis of these diseases.

## Future directions

It is likely that the role of Fe–S proteins will become increasingly recognized as key to metabolism and regulation of cellular responses as more key Fe–S proteins are identified and their roles are revealed. It is quite possible that Fe–S proteins represent a repository of energy stored in the form of reducing power, and that cells rely on this energy to maintain homeostasis. One could imagine that many pathways involved in cellular growth and regulation could be disrupted if a Fe–S cofactor becomes oxidized, leading to its dysfunction through changed conformation and/or charge interactions.

Iron sulfur cofactors evolved early in evolution, and the iron sulfur origin of life theory is a viable and attractive hypothesis (Boyd et al. 2017; Wächtershäuser 2007). By discovering direct protein- protein interactions between proteins in the Fe–S biogenesis pathways, it is likely that an elaborate distribution system for Fe–S cofactors will emerge that simultaneously ensures safe transfer and delivery to target proteins that need Fe–S cofactors for function, structure, or sensing. By following leads provided by direct interactions with proteins such as HSC20, NFU, ISCA, glutaredoxins and other proteins implicated in the process, the process will ultimately be sorted out, revealing exciting surprises and insights into growth of mammalian cells and tissues, and regulation of their functions.
